# Comparison of Mortality and Morbidity in Patients Undergoing Aortic Valve Replacement With or Without Aortic Root Enlargement

**DOI:** 10.7759/cureus.113852

**Published:** 2026-08-03

**Authors:** Surya SatyaGopal Palanki, Pramodh Reddy Jaggannagari, Madhusudan Kummari, Praveen Dontineni, Kaladhar Bomma, Amaresh Rao

**Affiliations:** 1 Cardiovascular and Thoracic Surgery, Nizam’s Institute of Medical Sciences, Hyderabad, IND; 2 Cardiothoracic Surgery, Nizam’s Institute of Medical Sciences, Hyderabad, IND

**Keywords:** aortic root enlargement, patient-prosthesis mismatch, small aortic annulus, surgical aortic valve replacement (savr), valve hemodynamics

## Abstract

Background

Surgical aortic valve replacement (SAVR) remains the standard treatment for many patients with severe aortic valve disease. Implantation of a small prosthetic valve may result in prosthesis-patient mismatch (PPM), which adversely affects postoperative valve hemodynamics and long-term outcomes. Aortic root enlargement (ARE) has been proposed as a technique to facilitate the implantation of larger prosthetic valves; however, evidence from Indian populations remains limited.

Methodology

This prospective, observational study was conducted at Nizam’s Institute of Medical Sciences, Hyderabad, over one year (2024-2025). A total of 40 patients undergoing AVR were included, of whom 22 underwent aortic valve replacement (AVR) alone, and 18 underwent AVR with ARE. Data were collected retrospectively and prospectively. Inclusion criteria comprised patients with moderate/severe symptomatic aortic stenosis or chronic severe aortic regurgitation undergoing elective SAVR. Exclusion criteria included active infection, prior prosthetic valves, or significant surgical risks. Data on demographics, preoperative variables, intraoperative details, and postoperative outcomes were collected using structured formats. Effective orifice area index was calculated to grade PPM severity. Statistical analysis included t-tests, chi-square test, Mann-Whitney U test, Kaplan-Meier survival, and logistic regression, with significance set at p-values <0.05.

Results

The median age of study subjects (n = 40) was 65.1 years (interquartile range = 53.2-71.3), with a female predominance (55%). Common comorbidities were hypertension (22/40, 55%), dyslipidemia (19/40, 47.5%), and diabetes mellitus (9/40, 22.5%). Preoperatively, 26 (65%) patients were categorized as New York Heart Association class III. Aortic stenosis (13/40, 32.5%) and mixed lesions (24/40, 60%) were the main pathologies. The mean implanted valve size was smaller in the ARE group than in the AVR group (21.2 mm vs. 23.5 mm; t(38) = 2.05, p = 0.047). Cardiopulmonary bypass (CPB) and aortic cross-clamp times were significantly longer in the ARE group (CPB: U = 107, p < 0.05; cross-clamp: t(38) = −2.07, p < 0.05). Postoperative mortality was comparable between groups (2/40, 5%; χ²(1) = 0.021, p = 0.884). The ARE group had a longer postoperative length of stay (median = 13 vs. 8 days; U = 80, p < 0.001). PPM occurred less frequently after ARE (χ²(3) = 9.045, p = 0.028); 26 patients (65%) had no PPM, and severe PPM was observed only in the AVR group. Kaplan-Meier analysis showed no difference in survival between groups over six months. Acute kidney injury was strongly associated with mortality (χ²(1) = 25.97, p = 0.004). Logistic regression identified female gender and lower effective orifice area as significant predictors for undergoing ARE.

Conclusions

This study highlights that ARE during AVR appears to be a safe adjunct procedure with comparable operative mortality or major complications in short-term follow-up. It effectively reduces the risk of PPM and allows implantation of larger prosthetic valves, which is particularly relevant in populations with smaller annuli. Although associated with longer operative and recovery times, ARE provides hemodynamic benefits without compromising short-term survival. These findings support the selective use of ARE in patients at risk for PPM to optimize surgical outcomes.

## Introduction

Aortic valve disease, particularly aortic stenosis (AS), remains an important contributor to cardiovascular morbidity and mortality worldwide. In high-income countries, degenerative calcific AS has become the predominant form of valvular heart disease because of increasing life expectancy and aging populations. In contrast, rheumatic heart disease (RHD) continues to impose a substantial burden in low- and middle-income countries, where disparities in healthcare access contribute to delayed diagnosis and treatment. Globally, more than 40 million people were affected by RHD in 2019, with the greatest burden occurring among socioeconomically disadvantaged populations [1]. In India, the epidemiology of aortic valve disease is gradually changing. Although rheumatic disease remains prevalent, degenerative AS now accounts for approximately 58-65% of isolated aortic valve lesions, while bicuspid aortic valve (BAV) contributes 25-34% and rheumatic pathology accounts for only 2-3% of cases. Indian patients also tend to present at a younger age than their counterparts in Western countries, with more than 60% being younger than 60 years, creating unique challenges in selecting the most appropriate treatment strategy [2].

Definitive management of severe AS primarily consists of surgical aortic valve replacement (SAVR) or transcatheter aortic valve replacement (TAVR). Over the past decade, TAVR has transformed the treatment of elderly and high-risk patients and has shown excellent clinical outcomes in appropriately selected populations. Nevertheless, its adoption in India remains constrained by procedural costs, limited reimbursement, and the need for specialized infrastructure and expertise [3]. Consequently, SAVR continues to be the standard treatment for most low- and intermediate-risk patients as well as those with complex valve anatomy. Prosthetic valve selection remains an important determinant of long-term outcomes. Mechanical valves provide excellent durability but necessitate lifelong anticoagulation, whereas bioprosthetic valves avoid chronic anticoagulation at the expense of structural degeneration over time. Although newer polymeric prosthetic valves have shown encouraging early results, they have not yet been incorporated into routine clinical practice [4,5].

One of the important challenges following SAVR is prosthesis-patient mismatch (PPM), which occurs when the effective orifice area (EOA) of the implanted prosthetic valve is disproportionately small relative to the patient’s body surface area, resulting in persistently elevated postoperative transvalvular gradients and less favorable hemodynamic performance. Patients with smaller aortic annuli, larger body surface area, and advanced age are particularly susceptible to developing PPM. Previous studies have demonstrated that PPM may occur in up to 52% of patients receiving small prosthetic valves (≤21 mm), emphasizing the importance of optimal prosthesis sizing during valve replacement [6]. Aortic root enlargement (ARE) has therefore emerged as an important adjunctive surgical technique that enlarges the native annulus, facilitates implantation of a larger prosthetic valve, and consequently reduces the likelihood of PPM. Contemporary evidence suggests that ARE is performed in approximately 15% of SAVR procedures and achieves short- and long-term outcomes comparable to isolated SAVR when performed in appropriate patients [7].

Although TAVR has broadened therapeutic options for patients with severe aortic valve disease, particularly those considered at high operative risk, SAVR remains the principal treatment modality in India because of financial limitations, restricted availability, and limited access to advanced structural heart programs. The early Indian experience reported by Gunasekaran et al. demonstrated the feasibility and favorable clinical outcomes of TAVR in selected patients while simultaneously underscoring the continuing importance of SAVR in routine cardiac surgical practice [8]. Among patients undergoing SAVR, PPM remains a clinically important concern, especially in individuals with a small aortic annulus, a situation frequently encountered in the Indian population. ARE has therefore gained increasing acceptance as an effective strategy to permit implantation of larger prosthetic valves and improve postoperative valve hemodynamics. Rocha et al. demonstrated that ARE significantly reduced the incidence of PPM without increasing operative mortality or major postoperative complications [9]. Likewise, Yousef et al. reported that incorporation of ARE during SAVR produced survival outcomes comparable to isolated AVR while improving postoperative valve performance and potentially facilitating future valve-in-valve interventions [10]. Despite these encouraging findings, evidence regarding the role of ARE in Indian patients remains limited, highlighting the need for further evaluation in this population.

Recent international studies have further expanded the understanding of the benefits and potential limitations of ARE. Hawkins et al. observed that increasing use of annular enlargement was associated with lower rates of PPM and improved suitability for future valve-in-valve procedures; however, they also reported higher adjusted morbidity and mortality, emphasizing the importance of appropriate patient selection and surgical expertise [11]. Mehaffey et al. demonstrated that elderly patients undergoing ARE experienced a greater incidence of early postoperative complications, although long-term survival appeared to favor annular enlargement beyond three years, suggesting that the advantages of preventing PPM may become more evident with extended follow-up [12]. Fallon et al. showed that PPM remains a common complication after SAVR and is associated with poorer long-term survival, increased heart failure readmissions, and a higher requirement for repeat aortic valve interventions, reinforcing the importance of strategies that minimize postoperative mismatch [13].

Additional evidence also supports the routine use of annular enlargement in appropriately selected patients. Castro et al. demonstrated that enlargement of the small aortic root can be performed with minimal additional operative risk while substantially reducing the incidence of PPM through implantation of larger prosthetic valves and improved postoperative hemodynamics [14]. Similarly, Dhareshwar et al. reported favorable perioperative outcomes with ARE and highlighted its value in patients with small aortic annuli who are particularly susceptible to PPM [15]. Furthermore, Brogan et al. showed that adequate relief of valvular obstruction is closely associated with postoperative functional improvement in patients with severe AS and low transvalvular gradients, underscoring the importance of achieving optimal valve hemodynamics during aortic valve replacement (AVR) [16]. Therefore, the present study aimed primarily to compare PPM reduction and valve hemodynamics, and secondarily to assess perioperative morbidity, mortality, and short-term clinical outcomes in patients undergoing SAVR with versus without ARE at a tertiary care center in India.

## Materials and methods

Study design and setting

This single-center, longitudinal, observational study, incorporating both retrospective and prospective designs, was conducted at the Department of Cardiovascular and Thoracic Surgery, Nizam’s Institute of Medical Sciences (NIMS), Hyderabad, Telangana, India.

Study population

Patients undergoing elective SAVR during the study period (2023-2025) and patients who underwent SAVR between January 2020 and December 2022 (retrospective) were considered for inclusion in the study.

Inclusion and exclusion criteria

Adult patients with moderate-to-severe symptomatic AS or chronic severe aortic regurgitation undergoing elective SAVR according to contemporary guideline recommendations were included. Patients with pre-existing prosthetic valves or annuloplasty devices in another cardiac position, active infective endocarditis, active systemic infection, ascending aortic aneurysm or dissection requiring circulatory arrest, porcelain aorta, hostile mediastinum, severe pulmonary arterial hypertension (systolic pulmonary artery pressure >60 mmHg), life expectancy of less than two years, advanced renal dysfunction requiring dialysis or estimated glomerular filtration rate <30 mL/minute/1.73 m², or those undergoing emergency surgery were excluded.

Sample size

The sample size was estimated using the formula (n = (Z×σ/E)^2^) for estimating a population mean based on the expected transvalvular pressure gradient reported in previous literature (mean = 22 mmHg; standard deviation = 5 mmHg), assuming an absolute precision of 1.6 mmHg and a 95% confidence interval (Z = 1.96). The calculated minimum sample size was 38 patients; therefore, 40 patients were enrolled in the study. Purposive sampling technique was employed in this study.

Methodology

After obtaining approval from the Institutional Ethics Committee (approval number: 1897/2025), an observational study incorporating both retrospective and prospective designs was conducted in the Department of Cardiovascular and Thoracic Surgery, NIMS, Hyderabad, India. A self-structured questionnaire (Appendix) was developed after review of the relevant literature and institutional practice guidelines and included questions regarding demographic characteristics, clinical findings, echocardiographic parameters, intraoperative variables, and postoperative outcomes. A pilot study was conducted among 10% of subjects (n = 5), and necessary changes were made in the questionnaire. Sampling was done purposively among 40 patients who met the inclusion criteria; patients with a small aortic root and requiring ARE were allocated to the SAVR + ARE group, and the remaining were allocated to the SAVR group. Data from patients operated on before January 2023 were obtained retrospectively from hospital case records, operative notes, and follow-up documentation, whereas patients undergoing surgery from January 2023 onward were enrolled prospectively and followed during hospitalization and subsequent outpatient visits. Written informed consent was obtained from all prospectively enrolled participants. Patients were followed for six months after surgery to assess postoperative clinical status, valve hemodynamics, and major adverse outcomes.

Surgical technique

Surgery was performed through a median sternotomy with cardiopulmonary bypass (CPB) using moderate systemic hypothermia (28-30°C), topical myocardial cooling with ice slush, and intermittent antegrade cold crystalloid cardioplegia administered through the aortic root or directly into the coronary ostia depending on aortic valve competence. Following an oblique (“hockey-stick”) aortotomy, the diseased valve was excised, the annulus was thoroughly debrided, and prosthetic valve sizing was performed using manufacturer-specific valve sizers. In patients requiring ARE, the aortotomy was extended through the non-coronary sinus across the aortic annulus into the anterior fibrous mitro-aortic curtain approximately 10-15 mm below the annulus. A tear-drop-shaped glutaraldehyde-treated Periti Plus™ bovine pericardial patch (Meril) was used to reconstruct the enlarged annulus using continuous 4-0 polypropylene sutures, allowing implantation of a prosthetic valve one or two sizes larger than initially measured. Valve sutures were subsequently placed circumferentially through the reconstructed annulus and prosthesis, followed by completion of valve implantation and closure of the aortotomy after appropriate trimming of the pericardial patch to maintain normal aortic root geometry.

Perioperative management protocol

Preoperative and postoperative transthoracic echocardiographic examinations were reviewed from the institutional electronic medical record system. Preoperative assessment included left ventricular ejection fraction (LVEF), aortic valve area, peak aortic jet velocity, and mean transvalvular pressure gradient. Postoperative echocardiography was used to evaluate prosthetic valve function, transvalvular gradients, EOA, and indexed effective orifice area (iEOA). PPM was assessed by dividing the published in vivo EOA of the implanted prosthetic valve by the patient's body surface area. PPM was classified as absent (iEOA >0.85 cm²/m²), moderate (iEOA = 0.65-0.85 cm²/m²), or severe (iEOA <0.65 cm²/m²) according to the criteria proposed by Pibarot and Dumesnil [6].

Statistical analysis

Statistical analysis was performed using SPSS Statistics for Windows, version 26.0 (IBM Corp., Armonk, NY, USA). Continuous variables are presented as mean ± standard deviation (SD) for normally distributed data or median (interquartile range (IQR)) for non-normally distributed data, while categorical variables are expressed as number (percentage). Comparisons between the isolated SAVR and SAVR + ARE groups were performed using the independent-samples Student’s t-test for normally distributed continuous variables, the Mann-Whitney U test for non-normally distributed variables, and the chi-square or Fisher’s exact test for categorical variables, as appropriate. Logistic regression analysis with backward elimination (entry criterion p < 0.20) was used to identify independent predictors of operative mortality. Short-term survival was evaluated using Kaplan-Meier analysis, and survival curves were compared using the log-rank test. A two-sided p-value <0.05 was considered statistically significant.

## Results

The present study was conducted among 40 patients who underwent SAVR in NIMS, Hyderabad. As shown in Table 1, the study cohort had a median age of 65.14 years (IQR = 53.2-71.3 years). Females constituted the majority of the study population, accounting for 55% of the participants.

**Table 1:** General information of the study population (N = 40).

Variable		Frequency
Age (years)		65.14 (53.2–71.3)
Gender	Female	22 (55%)
	Male	18 (45%)

Table 2 summarizes the preoperative characteristics of the study participants. The median body surface area was 2.02 m² (IQR = 1.79-2.14 m²). The majority of patients (65%) were categorized as New York Heart Association (NYHA) functional class III, while 6 (15%) each were in NYHA class II and class IV. Hypertension was present in 55% of the cohort, and 47.5% had dyslipidemia. Mixed aortic valve disease was the most common valvular pathology, observed in 60% of patients, followed by isolated aortic stenosis in 32.5%. The median preoperative transvalvular pressure gradient was 24.15 mmHg (IQR = 11.23-35.47 mmHg), and the median aortic root dimension was 0.78 cm (IQR = 0.62-0.89 cm). The mean preoperative LVEF was 50.8 ± 12.7%. The mean EOA and iEOA were 0.8 ± 0.5 cm² and 0.4 ± 0.3 cm²/m², respectively. The mean Society of Thoracic Surgeons (STS) predicted risk of mortality score was 1.7 ± 1.1.

**Table 2:** Distribution of preoperative details of the study participants (N = 40). *: Median (IQR) STS: Society of Thoracic Surgeons; IQR: interquartile range

Variable		Frequency, n (%)
Body surface area (m²)		2.02 (1.79–2.14)*
New York Heart Association functional class	1	2 (5%)
	2	6 (15%)
	3	26 (65%)
	4	6 (15%)
Associated comorbidities	Hypertension	22 (55%)
	Hyperlipidemia	19 (47.5%)
	Diabetes mellitus	9 (22.5%)
	Peripheral vascular disease	6 (15%)
	Chronic obstructive pulmonary disease	4 (10%)
	Chronic kidney disease on hemodialysis	2 (5%)
Type of aortic valve lesion	Stenosis	13 (32.5%)
	Insufficiency	3 (7.5%)
	Mixed	24 (60%)
Transvalvular pressure gradient (mmHg)		24.15 (11.23–35.47)*
Aortic root dimensions (cm)		0.78 (0.62–0.89)*
Ejection fraction (%)		50.8 ± 12.7
Effective orifice area (cm²)		0.8 ± 0.5
Effective orifice area index (cm²/m²)		0.4 ± 0.3
STS-predicted risk of mortality		1.7 ± 1.1

Table 3 shows the intraoperative details of study subjects. The mean prosthetic valve size was 22.3 ± 2.1 mm, and the majority 34 (85%) were mechanical valves. The mean cross-clamp time during the surgery was 73.2 ± 23.7 minutes, and the mean CPB time was 133 (105-177) minutes. Among 22 (55%) study subjects, standard AVR was performed, whereas among 18 (45%) patients, AVR + ARE was performed.

**Table 3:** Distribution of intraoperative details of the study subjects (N = 40). *: Median (IQR). AVR: aortic valve replacement; ARE: aortic root enlargement; IQR: interquartile range

Variable		Frequency, n (%)
Valve size (mm)		22.3 ± 2.1
Valve type	Mechanical	34 (85%)
	Bioprosthetic	6 (15%)
Cross-clamp time (minutes)		73.2 ± 23.7
Cardiopulmonary bypass time (minutes)		133 (105–177)*
Type of surgery	AVR	22 (55%)
	AVR + ARE	18 (45%)

Table 4 shows the postoperative details. Overall, 5% of subjects died postoperatively. The median length of stay in the hospital was 7 (5-10) days, and 35% of patients were transfused with blood products during the postoperative period. About 3 (7.5%) patients suffered from stroke and acute kidney injury. Overall, 7 (17.5%) patients needed prolonged ventilation (>24 hours), and among 12.5% of patients, an endocardial permanent pacemaker was implanted. About 11 (27.5%) patients suffered from atrial fibrillation during the postoperative period, and 12.5% of patients needed hospital readmission within 30 days. About 2 (5%) patients needed reoperation. The postoperative median trans-implanted valve pressure gradient was 11.9 (8.5-14.9) mm Hg. The postoperative median EOA was 1.7 (1.5-1.9) cm² and iEOA was 0.83 (0.71-0.87) cm²/m². The majority (26, 65%) of the patients had no PPM, whereas 8 (20%) had mild PPM, followed by 4 (10%) with moderate PPM, and 2 (5%) with severe PPM.

**Table 4:** Distribution of postoperative details of the study subjects (N = 40). *: Median (IQR). IQR: interquartile range

Variable		Frequency, n (%)
Operative mortality	Alive	38 (95%)
	Dead	2 (5%)
Length of stay (days)		7 (5–10))
Blood product transfusion		14 (35%)
Stroke		3 (7.5%)
Acute kidney injury		3 (7.5%)
Prolonged ventilation (>24 hours)		7 (17.5%)
Permanent pacemaker implantation		5 (12.5%)
Atrial fibrillation		11 (27.5%)
30-day readmission		5 (12.5%)
Reoperation (for any cause)		2 (5%)
Trans-implanted valve pressure gradient (mmHg)		11.9 (8.5–14.9)*
Effective orifice area (cm²)		1.7 (1.5–1.9)*
Indexed effective orifice area (cm²/m²)		0.83 (0.71–0.87)*
Prosthesis-patient mismatch	None	26 (65%)
	Mild	8 (20%)
	Moderate	4 (10%)
	Severe	2 (5%)

**Figure 1:**
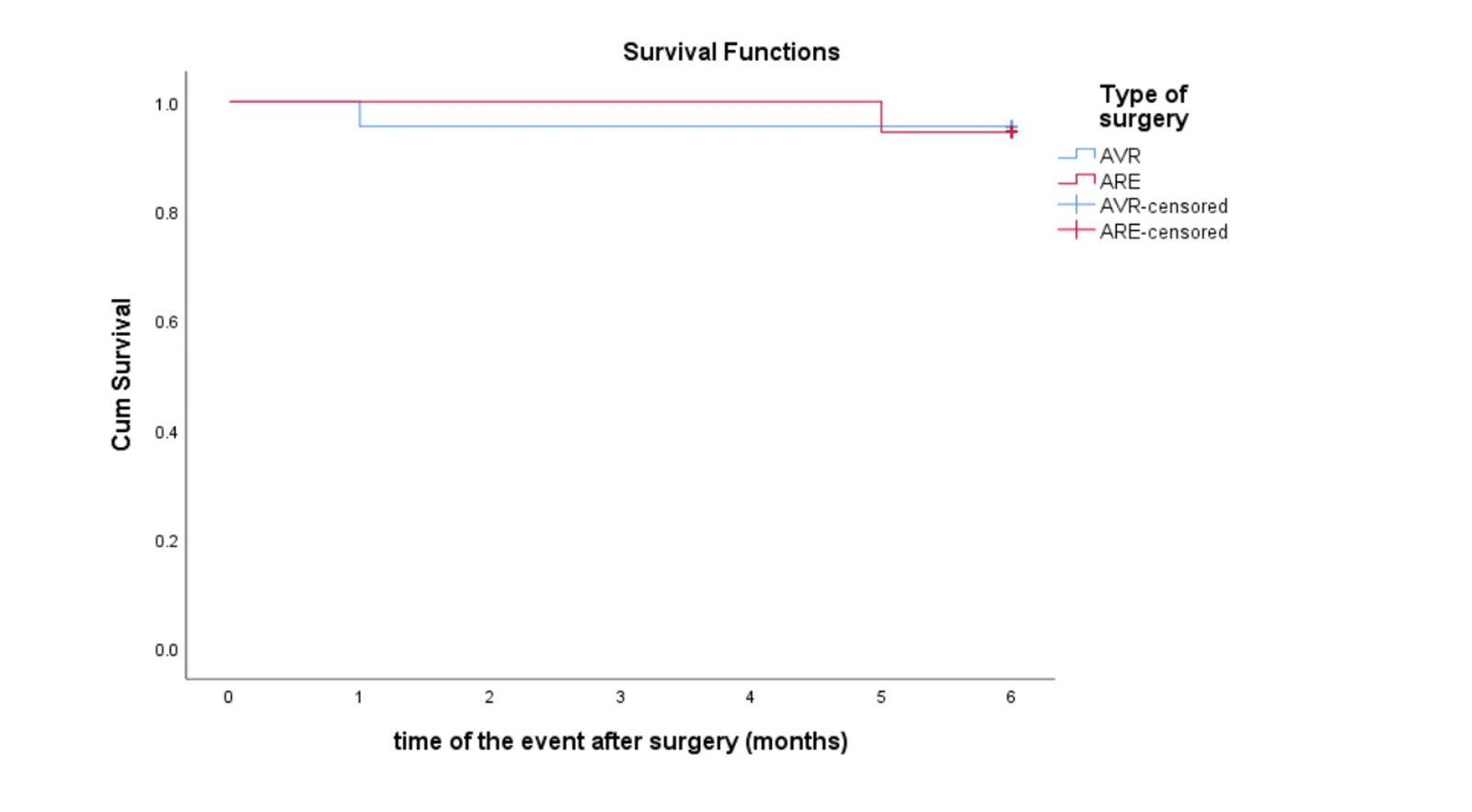
Comparison of short-term survival after AVR and ARE surgeries using Kaplan-Meier analysis. AVR: aortic valve replacement; ARE: aortic root enlargement

Kaplan-Meier analysis demonstrated high short-term survival in both groups over six months. Survival remained comparable between isolated AVR and AVR with ARE, with no meaningful separation of the curves during follow-up (Figure 1).

Table 5 shows that females significantly (p = 0.04) underwent ARE more compared to AVR, whereas other preoperative factors were not significantly associated with the type of surgery.

**Table 5:** Association between preoperative factors and type of surgery among the study subjects (N = 40). *: Median (IQR); Mann-Whitney U test = U value (n1, n2); ^#^: t-test = t-value (df). AVR: aortic valve replacement; ARE: aortic root enlargement; NYHA: New York Heart Association; COPD: chronic obstructive pulmonary disease; STS: Society of Thoracic Surgeons

Preoperative factors		Type of surgery		Total	X² (df)	P-value
		AVR	AVR + ARE			
Age		54.5 (44.5–60)	56 (57–73)	65.5 (53–71.5)	180 (22, 18)*	0.635
Gender	Female	9 (40.9)	13 (59.1)	22 (55)	3.922 (1)	0.047
	Male	13 (72.2)	5 (27.8)	18 (45)		
Body surface area		1.99 (1.75–2.13)	1.97 (1.78–2.10)	2.02 (1.79–2.14)*	175 (22, 18)*	0.539
NYHA functional class	1	1 (50)	1 (50)	2 (5)	0.425 (3)	0.935
	2	4 (66.7)	2 (33.3)	6 (15)		
	3	14 (53.8)	12 (46.2)	26 (65)		
	4	3 (50)	3 (50)	6 (15)		
Associated comorbidities	Hypertension	12 (54.5)	10 (45.5)	22 (55)	0.004 (1)	0.949
	Hyperlipidemia	10 (52.6)	9 (47.4)	19 (47.5)	0.082 (1)	0.775
	Diabetes mellitus	4 (44.4)	5 (55.6)	9 (22.5)	0.523 (1)	0.470
	Peripheral vascular disease	4 (66.7)	2 (33.3)	6 (15)	0.388 (1)	0.533
	COPD	2 (50)	2 (50)	4 (10)	0.045 (1)	0.832
	Dialysis	1 (50)	1 (50)	2 (5)	0.021 (1)	0.884
Type of aortic valve lesion	Stenosis	6 (46.2)	7 (53.8)	13 (32.5)	0.684 (2)	0.710
	Insufficiency	2 (66.7)	1 (33.3)	3 (7.5)		
	Mixed	14 (58.3)	10 (41.7)	24 (60)		
Ejection fraction		51.5 ± 12.6	49.9 ± 13.1	50.8 ± 12.7	0.720 (38)^#^	0.476
Effective orifice area (cm²/m²)		0.81 ± 0.5	0.79 ± 0.4	0.8 ± 0.5	0.625 (38)^#^	0.536
Effective orifice area index (cm²/m²)		0.41 ± 0.3	0.4 ± 0.3	0.4 ± 0.3	0.475 (38)^#^	0.637
STS-predicted risk of mortality		1.7 ± 1.0	1.7 ± 1.1	1.7 ± 1.1	0.164 (38)^#^	0.871

Table 6 shows that the prosthetic valve size was significantly smaller among patients undergone AVR + ARE than their counterparts. Intraoperative factors such as cross-clamp time and CPB time were significantly higher among patients undergoing AVR + ARE, whereas valve type and aortic annular enlargement were not different between the two groups (p > 0.05).

**Table 6:** Association between intraoperative factors and type of surgery among the study subjects (N = 40). *: Median (IQR); Mann-Whitney U test = U value (n1, n2); ^#^: t-test = t-value (df). AVR: aortic valve replacement; ARE: aortic root enlargement

Intraoperative factors		Type of surgery		Total	X^2^ (df)	P-value
		AVR (n = 22)	AVR + ARE (n = 18)			
Valve size		23.5 ± 2.6	21.2 ± 1.9	22.3 ± 2.1	2.053 (38)^#^	0.047
Valve type	Mechanical	19 (55.9)	15 (44.1)	34 (85)	0.071 (1)	0.789
	Bioprosthetic	3 (50)	3 (50)	6 (15)		
Aortic annular enlargement		3 (37.5)	5 (62.5)	8 (20)	1.237 (1)	0.266
Cross-clamp time (minutes)		66.1 ± 25.5	75.7 ± 28.9	73.2 ± 23.7	2.074 (38)^#^	0.025
Cardiopulmonary bypass time (minutes)		121 (87–142)	134 (94–156)	133 (105–177) *	107 (22, 18)^*^	0.014

Table 7 shows the association between postoperative factors and the type of surgery among the study participants. Postoperative length of hospital stay was significantly longer (p < 0.00) in the AVR + ARE group, whereas PPM was significantly higher in the AVR group (p = 0.028). However, other postoperative factors were not significantly different between the two groups (p > 0.05).

**Table 7:** Association between postoperative factors and type of surgery among the study subjects (N = 40). *: Median (IQR); Mann-Whitney U test = U value (n1, n2); ^#^: t-test = t-value (df). AVR: aortic valve replacement; ARE: aortic root enlargement

Postoperative factors		Type of surgery		Total (%)	X^2^ (df)	P-value
		AVR (n = 22)	AVR + ARE (n = 18)			
Operative mortality		1 (50)	1 (50)	2 (5)	0.021 (1)	0.884
Length of stay (days)		8 (5–11)	13 (7–19)	8.5 (5–16)	80 (22, 18)*	<0.001
Blood product transfusion		7 (50)	7 (50)	14 (35)	0.218 (1)	0.641
Stroke		1 (50)	1 (50)	2 (5)	0.210 (1)	0.884
Acute kidney injury		2 (66.7)	1 (33.3)	3 (7.5)	0.178 (1)	0.673
Prolonged ventilation (>24 hours)		4 (57.1)	3 (42.9)	7 (17.5)	0.016 (1)	0.900
Permanent pacemaker implantation		3 (60)	2 (40)	5 (12.5)	0.058 (1)	0.810
Atrial fibrillation		6 (54.5)	5 (45.5)	11 (27.5)	0.001 (1)	0.972
30-day readmission		3 (60)	2 (40)	5 (12.5)	0.058 (1)	0.810
Reoperation (for any cause)		1 (50)	1 (50)	2 (5)	0.021 (1)	0.884
Trans-implanted valve pressure gradient		11.5 (9–15)	12 (8–15)	11.5 (8–16)	191 (22,18)^*^	0.857
Effective orifice area		1.3 ± 0.6	1.5 ± 0.8	1.5 ± 0.7	1.467 (38)^#^	0.135
Indexed effective orifice area		0.6 ± 0.4	0.7 ± 0.4	0.7 ± 0.4	1.369 (38)^#^	0.179
Prosthesis-patient mismatch	None	10 (38.5)	16 (61.5)	26 (65)	9.045 (3)	0.028
	Mild	6 (75)	2 (25)	8 (20)		
	Moderate	4 (100)	0 (0)	4 (10)		
	Severe	2 (100)	0 (0)	2 (5)		

Table 8 shows that patients who had acute kidney injury in the postoperative period had significantly (p = 0.004) increased mortality than their counterparts. Postoperative factors such as stroke and atrial fibrillation did not increase the mortality rates significantly (p > 0.05).

**Table 8:** Association between postoperative complications and survival outcome among the study participants (N = 40).

Postoperative complications		Mortality		Total (%)	X^2 ^(df)	P-value
		Yes	No			
Stroke	Yes	1 (50)	1 (50)	2 (5)	8.975 (1)	0.099
	No	1 (2.6)	37 (97.4)	38 (95)		
Acute kidney injury	Yes	2 (66.7)	1 (33.3)	3 (7.5)	25.965 (1)	0.004
	No	0 (0)	37 (100)	37 (32.5)		
Atrial fibrillation	Yes	2 (18.2)	9 (81.8)	11 (27.5)	5.550 (1)	0.071
	No	0 (0)	29 (100)	29 (72.5)		

Table 9 shows the association between PPM and EOA, where there was no significant (p = 0.435) association between mean EOA and PPM.

**Table 9:** Association of prosthesis-patient mismatch with effective orifice area among the study subjects (N = 40). *: Analysis of variance test.

Patient-prosthesis mismatch	Effective orifice area (mean ± SD)	F-value (P-value)*
None	1.4 ± 0.8	0.933 (0.435)
Mild	1.5 ± 0.7	
Moderate	1.3 ± 0.7	
Severe	1.4 ± 0.7	
Total	1.5 ± 0.7	

Table 10 shows that most covariates did not show a significant association with the type of surgery. Notably, gender was significant (p = 0.006), suggesting a strong protective effect for males (odds ratio (OR) = 0.037) to have ARE compared to females. Participants with less EOA were also significant (p = 0.045; OR = 10.06) and showed a strong association with ARE compared to their counterparts. Other variables, such as age, body surface area, type of aortic valve disease, and all procedural or postoperative variables, did not have an association with the type of surgery.

**Table 10:** Logistic regression models for predictors of type of surgery (AVR vs. AVR + ARE) (N = 40). EOA: effective orifice area; iEOA: indexed effective orifice area; CPB: cardiopulmonary bypass; PPM: prosthesis-patient mismatch; AVR: aortic valve replacement; ARE: aortic root enlargement

Covariate	OR (95% CI)	P-value
Age	1.022 (0.970–1.077)	0.409
Gender	0.037 (0.003–0.394)	0.006
Body surface area	8.971 (0.144–560.45)	0.298
Type of aortic valve disease	1.575 (0.704–3.526)	0.269
Transvalvular pressure gradient	0.988 (0.882–1.107)	0.831
Aortic root dimensions	0.715 (0.001–690.73)	0.924
Ejection fraction	1.005 (0.937–1.078)	0.890
EOA	10.06 (10.052–96.10)	0.045
iEOA	1.397 (0.054–36.282)	0.840
Valve size	1.331 (0.957–1.852)	0.090
Valve type	0.779 (0.103–5.914)	0.810
Aortic annular enlargement	1.117 (0.145–8.582)	0.915
Cross-clamp time	1.039 (0.960–1.124)	0.346
CPB time	0.987 (0.957–1.019)	0.432
Length of stay	0.606 (0.287–1.283)	0.191
Blood product transfusion	6.823 (0.042–1110.15)	0.460
Permanent pacemaker implantation	0.521 (0.001–737.59)	0.860
Atrial fibrillation	0.333 (0.001–175.96)	0.731
Trans-implanted valve pressure gradient	1.732 (0.661–4.535)	0.264
PPM	0.003 (0.001–1.266)	0.060

## Discussion

The present study was conducted at NIMS, Hyderabad, to evaluate and compare outcomes following AVR with and without ARE, with particular emphasis on PPM, perioperative outcomes, and factors associated with the need for ARE.

Baseline characteristics

The median age of our cohort was 65 (53.2-71.3) years, which was lower than that reported by Gunasekaran et al. [8], Yousef et al. [10], Hawkins et al. [11], Mehaffey et al. [12], Fallon et al. [13], Dhareshwar et al [15], Kumar et al. [17], and Vriesendorp et al. [18]. Age was not significantly associated with the type of surgery in the present study, consistent with the findings of Rao et al. [7] and Castro et al. [14]. In contrast, Yousef et al. [10], Hawkins et al. [11], Mehaffey et al. [12], and Dhareshwar et al [15] reported a significant association between age and the type of surgery performed. Females constituted 55% (n = 22) of the study population, which was slightly higher than that reported by Fallon et al. [13] and Kumar et al. [17]. The median body surface area was 2.02 (1.79-2.14) m², comparable to the findings reported by Fallon et al. [13] and Vriesendorp et al. [18]. Hypertension was the most common comorbidity, affecting 22 (55%) patients, similar to the findings reported by Gunasekaran et al. [8], Hawkins et al. [11], and Fallon et al. [13]. Diabetes mellitus was present in 9 (22.5%) patients, a lower prevalence than that reported by Gunasekaran et al. [8] and Vriesendorp et al. [18]. The majority of patients (26, 65%) were in NYHA functional class III, which is comparable to the proportions reported by Fallon et al. [13] and Kumar et al. [17].

Preoperative echocardiographic and risk profile

The mean LVEF in the present study was 50.8 ± 12.7%, which was similar to the findings of Gunasekaran et al. [8] but lower than that reported by Vriesendorp et al. [18]. The mean EOA and iEOA were 0.8 ± 0.5 cm² and 0.4 ± 0.3 cm²/m², respectively, closely matching the values reported by Vriesendorp et al. [18]. The mean transvalvular pressure gradient was 24.15 mmHg, which was higher than that reported by Brogan et al. [16] but lower than that observed by Pereira et al. [19]. The mean STS-predicted risk of mortality was 1.7 ± 1.1%, lower than that reported by Kumar et al. [17] and Vriesendorp et al. [18].

Operative characteristics

Approximately 8 (20%) patients required ARE, which is considerably higher than the 3.2% reported by Fallon et al. [13]. Patients undergoing ARE had significantly longer aortic cross-clamp times, consistent with the findings reported by Rao et al. [7], Rocha et al. [9], Hawkins et al. [11], Castro et al. [14], Dhareshwar et al. [15], and Prakash et al. [20]. Similarly, CPB times were significantly longer in the ARE group, a finding also reported by Rao et al. [7], Rocha et al. [9], Hawkins et al. [11], Castro et al. [14], Dhareshwar et al [15], and Prakash et al. [20]. The increased operative duration associated with ARE is expected because enlargement procedures require additional annular reconstruction and patch augmentation, thereby increasing procedural complexity.

Postoperative outcomes and complications

In the present study, operative mortality did not differ significantly between AVR and AVR + ARE groups, similar to the findings reported by Yousef et al. [10] and Dhareshwar et al [15]. However, Rocha et al. [9] and Hawkins et al. [11] reported higher mortality among patients undergoing ARE. Postoperative stroke occurred in 2 (5%) patients, comparable to the incidence reported by Vriesendorp et al. [18]. Stroke was not associated with the type of surgery, consistent with the findings reported by Rao et al. [7], Rocha et al. [9], Yousef et al. [10], Hawkins et al. [11], Castro et al. [14], and Dhareshwar et al. [15]. Atrial fibrillation occurred in 27.5% of patients, which was higher than the rates reported by Gunasekaran et al. [8] and Vriesendorp et al. [18]. However, atrial fibrillation was not significantly associated with the type of surgery, consistent with reports by Rao et al. [7], Rocha et al. [9], Yousef et al. [10], and Prakash et al. [20]. Permanent pacemaker implantation was required in 5 (12.5%) patients and was not associated with the type of surgery. Similar findings have been reported by Rao et al. [7], Yousef et al. [10], and Dhareshwar et al [15], whereas Rocha et al. [9] demonstrated a significant association. Renal injury was not associated with the type of surgery, consistent with the findings reported by Rao et al. [7] and Hawkins et al. [11], although Rocha et al. [9] and Yousef et al. [10] reported significant associations. Postoperative length of hospital stay was significantly longer in patients undergoing ARE, consistent with the findings reported by Yousef et al. [10], although Hawkins et al. [11] found no difference between groups. Thirty-day readmission, reoperation, and postoperative mean aortic valve gradient were also not associated with the type of surgery, similar to the findings reported by Yousef et al. [10]. A major finding of the present study was the significantly lower incidence of PPM among patients undergoing ARE. This finding supports the theoretical benefit of annular enlargement by permitting implantation of larger prosthetic valves and increasing the EOA. In contrast, Yousef et al. [10] and Hawkins et al. [11] reported no significant differences in PPM between groups. Overall survival at six months was 95%. Survival was 95.6% in the AVR group and 94.4% in the AVR + ARE group, indicating no significant difference between strategies. These survival rates compare favorably with those reported by Rao et al. [7], who observed approximately 90% survival at one year.

Predictors of aortic root enlargement

Female sex was significantly associated with undergoing ARE. Similar findings have been reported by Rocha et al. [9], Yousef et al. [10], Hawkins et al. [11], Mehaffey et al. [12], Castro et al. [14], and Dhareshwar et al. [15]. This observation likely reflects the smaller annular dimensions typically encountered in female patients. Body surface area was not associated with the type of surgery in the present study, similar to the findings reported by Rao et al. [7], Yousef et al. [10], Hawkins et al. [11], and Prakash et al. [20]. However, significant associations have been reported by Rocha et al. [9], Castro et al. [14], and Dhareshwar et al. [15]. NYHA functional class was not associated with the type of surgery, consistent with findings reported by Rao et al. [7], Castro et al. [14], Dhareshwar et al. [15], and Prakash et al. [20]. In contrast, Rocha et al. [9] reported a significant association. Similarly, EOA, iEOA, STS-predicted mortality, and valve type were not significantly associated with the type of surgery, findings that largely agree with previous reports [7,10,11,15]. Patients with smaller aortic valve sizes were significantly more likely to require ARE, similar to the findings reported by Yousef et al. [10], Castro et al. [14], and Prakash et al. [20]. Overall, the present study demonstrates that ARE can be performed safely with acceptable perioperative morbidity and mortality. Although ARE increases operative complexity and prolongs CPB and cross-clamp times, it substantially reduces the incidence of PPM without adversely affecting short-term survival.

Limitations

The findings of this study should be interpreted in light of several limitations. First, this was a single-center study conducted at a tertiary care referral institution, which may limit the generalizability of the results to other regions of India owing to variations in patient demographics, disease profiles, comorbidity burden, referral patterns, and access to healthcare services.

Second, the study included a retrospective component, which is prone to missing data and limited control of confounders. It combined cases from 2020-2022 (retrospective) with prospectively enrolled patients from 2023-2025, a period when perioperative practices, valve selection, and postoperative care may have changed. These temporal differences could introduce time-period bias, with outcomes reflecting evolving institutional protocols rather than the surgical strategy alone.

Third, the relatively short duration of follow-up restricted the assessment of long-term clinical outcomes, prosthetic valve durability, structural valve deterioration, and the long-term impact of PPM.

Fourth, the study enrolled 40 patients (22 SAVR, 18 SAVR + ARE); sample size targeted transvalvular gradient differences, not clinical outcomes. Therefore, it is underpowered to detect modest differences in perioperative mortality or rare complications, and non-significant findings may reflect Type II error.

Fifth, although multivariable logistic regression was used, residual confounding may persist; baseline annular size and other anatomical or clinical factors influencing the surgeon’s choice of ARE might be incompletely adjusted. Thus, the association between ARE, PPM, and outcomes remains hypothesis-generating, not causal.

Finally, the possibility of selection bias cannot be excluded, as referral and treatment practices in a high-volume tertiary care center such as NIMS may differ from those in community hospitals or smaller cardiac centers. Larger multicenter studies with longer follow-up durations are therefore warranted to validate these findings and better define the long-term benefits of ARE during AVR.

## Conclusions

In this longitudinal observational study comparing outcomes following AVR with and without ARE, patients undergoing ARE demonstrated significantly lower rates of PPM despite requiring longer CPB and aortic cross-clamp times. Postoperative valve hemodynamics were favorable across the cohort, with significant improvements in EOA and transvalvular gradients. Although perioperative complications such as atrial fibrillation, prolonged ventilation, stroke, pacemaker implantation, and acute kidney injury were observed, the overall morbidity and mortality rates remained acceptable, with a 95% six-month survival rate and no significant difference in survival between the AVR-only and AVR + ARE groups. Female sex and smaller preoperative EOA were significant predictors of undergoing ARE, while acute kidney injury emerged as an independent predictor of postoperative mortality. Importantly, PPM was significantly more common in patients undergoing AVR without ARE, underscoring the hemodynamic benefits of annular enlargement. These findings suggest that ARE was associated with acceptable perioperative morbidity and mortality, and short-term survival was similar between patients undergoing isolated AVR and AVR with ARE. However, given the limited sample size and event rates, these findings should be interpreted cautiously and cannot exclude clinically important differences in risk. Given the high prevalence of small aortic annuli among patients in the Indian subcontinent, ARE should be considered whenever anatomically feasible, as it may improve short-term clinical outcomes and enhance future treatment options, including valve-in-valve transcatheter interventions. However, larger multicenter studies with longer follow-up are required to confirm the safety profile of ARE and its impact on long-term clinical outcomes. ARE should be considered in patients with small aortic annuli, particularly in female patients, who were significantly more likely to require the procedure in this study. ARE facilitates the implantation of larger prosthetic valves, thereby improving the EOA and reducing the risk of PPM, which is associated with suboptimal postoperative hemodynamics. Although ARE offers important hemodynamic advantages, it is associated with significantly longer aortic cross-clamp and CPB times, highlighting the need for careful preoperative planning, appropriate patient selection, and meticulous intraoperative management to balance the benefits of annular enlargement against the increased operative complexity.

## Appendices

**Table 11:** Questionnaire used in the study.

Questionnaire	
Identification details: Name: Age (years): Sex: Preoperative data: Body surface area (m²): NYHA functional class: I, II, III, IV. Associated comorbidities: hypertension/diabetes mellitus/hyperlipidemia/chronic obstructive pulmonary disease/Others. Echocardiographic findings: Type of aortic valve lesion: Stenosis/Insufficiency/Mixed. Transvalvular pressure gradient (mmHg). Aortic root dimensions (cm): Ejection fraction (%): Effective orifice area (cm²): Effective orifice area index: (cm²/m²). Intraoperative date: Valve size (mm). Valve type: Mechanical/Bioprosthetic. Aortic annular enlargement: Yes/No. Cross-clamp time (minutes): Cardiopulmonary bypass time (minutes):	Postoperative date: Operative mortality: Alive/Dead. Length of stay (days): Blood product transfusion: Yes/No. Stroke: Yes/No. Acute kidney injury: Yes/No. Prolonged ventilation (>24 hours): Yes/No. Permanent pacemaker implantation: Yes/No. Atrial fibrillation: Yes/No. 30-day readmission: Yes/No. Reoperation (for any cause): Yes/No. Trans-implanted valve pressure gradient (9 mmHg): Effective orifice area (cm²): Indexed effective orifice area (cm²/m²): Prosthesis-patient mismatch: None/Mild/Moderate/Severe

## References

[1] Santangelo G, Bursi F, Faggiano A (2023). The global burden of valvular heart disease: from clinical epidemiology to management. J Clin Med.

[2] Sahu AK, Sagar P, Khanna R (2020). Etiology and distribution of isolated aortic stenosis in Indian patients - a study from a large tertiary care hospital in north India. Indian Heart J.

[3] Gupta P, Arora S, Qamar A, Gupta M, Seth A (2020). Current status of transcatheter aortic valve replacement in India. Cardiovasc Diagn Ther.

[4] Manji RA, Menkis AH, Ekser B, Cooper DK (2012). The future of bioprosthetic heart valves. Indian J Med Res.

[5] Agrawal A (2024). Transcatheter heart valves—an update on types of valves available, hardware characteristics, and patient selection: an Indian perspective. Indian J Clin Cardiol.

[6] Pibarot P, Dumesnil JG (2000). Hemodynamic and clinical impact of prosthesis-patient mismatch in the aortic valve position and its prevention. J Am Coll Cardiol.

[7] Rao V, Linick JA, Reardon MJ (2023). Early and late effects of aortic root enlargement: results from the Pericardial Surgical Aortic Valve Replacement Pivotal Trial: a multicenter, prospective clinical trial. JTCVS Open.

[8] Gunasekaran S, Sivaprakasam MC, PaulPandi VK (2018). Transcatheter aortic valve replacement in India-early experience, challenges, and outcomes from a single center. Indian Heart J.

[9] Rocha RV, Manlhiot C, Feindel CM, Yau TM, Mueller B, David TE, Ouzounian M (2018). Surgical enlargement of the aortic root does not increase the operative risk of aortic valve replacement. Circulation.

[10] Yousef S, Brown JA, Serna-Gallegos D (2023). Impact of aortic root enlargement on patients undergoing aortic valve replacement. Ann Thorac Surg.

[11] Hawkins RB, Beller JP, Mehaffey JH (2019). Incremental risk of annular enlargement: a multi-institutional cohort study. Ann Thorac Surg.

[12] Mehaffey JH, Hawkins RB, Wegermann ZK (2021). Aortic annular enlargement in the elderly: short and long-term outcomes in the United States. Ann Thorac Surg.

[13] Fallon JM, DeSimone JP, Brennan JM (2018). The incidence and consequence of prosthesis-patient mismatch after surgical aortic valve replacement. Ann Thorac Surg.

[14] Castro LJ, Arcidi JM Jr, Fisher AL, Gaudiani VA (2002). Routine enlargement of the small aortic root: a preventive strategy to minimize mismatch. Ann Thorac Surg.

[15] Dhareshwar J, Sundt TM 3rd, Dearani JA, Schaff HV, Cook DJ, Orszulak TA (2007). Aortic root enlargement: what are the operative risks?. J Thorac Cardiovasc Surg.

[16] Brogan WC 3rd, Grayburn PA, Lange RA, Hillis LD (1993). Prognosis after valve replacement in patients with severe aortic stenosis and a low transvalvular pressure gradient. J Am Coll Cardiol.

[17] Kumar V, Sengottuvelu G, Singh VP, Rastogi V, Seth A (2022). Transcatheter aortic valve implantation for severe bicuspid aortic stenosis - 2 years follow up experience from India. Front Cardiovasc Med.

[18] Vriesendorp MD, de Lind van Wijngaarden RA, Klautz RJ (2020). Concomitant aortic root enlargement is perhaps safe, but is it also effective?. Eur J Cardiothorac Surg.

[19] Pereira JJ, Lauer MS, Bashir M (2002). Survival after aortic valve replacement for severe aortic stenosis with low transvalvular gradients and severe left ventricular dysfunction. J Am Coll Cardiol.

[20] Prakash S, Agarwal S, Dutta N, Satsangi DK (2010). A comparative study of surgical treatment of small aortic root with or without aortic root enlargement using a single prosthesis type. J Cardiovasc Med (Hagerstown).

